# Novel *BMP4* Truncations Resulted in Opposite Ocular Anomalies: Pathologic Myopia Rather Than Microphthalmia

**DOI:** 10.3389/fcell.2021.769636

**Published:** 2021-12-01

**Authors:** Yi Jiang, Jiamin Ouyang, Xueqing Li, Yingwei Wang, Lin Zhou, Shiqiang Li, Xiaoyun Jia, Xueshan Xiao, Wenmin Sun, Panfeng Wang, Qingjiong Zhang

**Affiliations:** ^1^State Key Laboratory of Ophthalmology, Zhongshan Ophthalmic Center, Sun Yat-sen University, Guangzhou, China; ^2^Department of Ophthalmology, West China Hospital, Sichuan University, Chengdu, China

**Keywords:** pathologic myopia, early-onset myopia, *BMP4*, truncation variants, microphthalmia

## Abstract

*BMP4* variants have been reported to be associated with syndromic microphthalmia (MCOPS6, OMIM 607932). This study aims to describe *BMP4* truncation mutations contributing to a novel phenotype in eight patients from four Chinese families. In this study, *BMP4* variants were collected from a large dataset from in-house exome sequencing. Candidate variants were filtered by multiple *in silico* tools as well as comparison with data from multiple databases. Potential pathogenic variants were further confirmed by Sanger sequencing and cosegregation analysis. Four novel truncation variants in *BMP4* were detected in four out of 7,314 unrelated probands with different eye conditions. These four mutations in the four families solely cosegregated in all eight patients with a specific form of pathologic myopia, characterized by significantly extended axial length, posterior staphyloma, macula patchy, chorioretinal atrophy, myopic optic neuropathy or glaucoma, vitreous opacity, and unique peripheral snow-grain retinopathy. The extreme rarity of the truncations in *BMP4* (classified as intolerant in the gnomAD database, pLI = 0.96), the exclusive presence of these variants in the four families with pathologic myopia, variants fully co-segregated with the same specific phenotypes in eight patients from the four families, and the association of the pathogenicity of truncations with syndromic microphthalmia in previous studies, all support a novel association of *BMP4* truncations with a specific form of pathologic myopia. The data presented in this study demonstrated that heterozygous *BMP4* truncations contributed to a novel phenotype: pathologic myopia rather than microphthalmia. Mutations in the same gene resulting in both high myopia and microphthalmia have been observed for a few other genes like *FZD5* and *PAX6*, suggesting bidirectional roles of these genes in early ocular development. Further studies are expected to elucidate the molecular mechanism of the bidirectional regulation.

## Introduction

Pathologic myopia is characterized by posterior staphyloma, fundus degenerative changes, and abnormal corrected visual acuity. Pathologic myopia usually belongs to a subgroup of high myopia, which is defined as an axial length of 26 mm or more (Ohno-Matsui et al., 2016; Sankaridurg et al., 2021; Spaide et al., 2021). The complications associated with pathologic myopia are among the first to third common causes of legal blindness worldwide (Wong et al., 2014; Sankaridurg et al., 2021). Genetic defects play a major role in the development of pathologic myopia or high myopia, and among these defects, at least 28 loci or genes have been reported to contribute to non-syndromic forms, while variants in a number of genes are known to cause syndromic forms. However, the genetic defects for most cases of high myopia or pathologic myopia are still unknown, and the identification of additional implicating genes may enrich our understanding of the pathogenesis and facilitate the prevention and management of these conditions (Zhang, 2021).

Comparative exome sequencing has been used to detect genetic factors contributing to retinitis pigmentosa and glaucoma in our previous studies (Sun et al., 2019; Yi et al., 2020). Using similar strategy, four truncation variants in *BMP4* (OMIM 112262) were detected in four unrelated families with pathologic myopia. These variants were confirmed by Sanger sequencing and cosegregated with pathologic myopia in eight patients from four families but in none of the unaffected individuals or any in-house controls, suggesting that *BMP4* may be an important factor for pathologic myopia. *BMP4* plays a vital regulatory role in embryonic development (Hogan, 1996) and truncations in this gene are extremely rare and intolerant [gnomAD, probability of being loss-of-function intolerant (pLI) = 0.96; Exp. 14.2 with obs. 1]. Previously, truncations in *BMP4* were reported to cause microphthalmia, anophthalmia, and coloboma (MAC) (Reis et al., 2011) and anterior segment dysgenesis (ASGD) (Takenouchi et al., 2013), phenotypes that in contrast to those of pathologic myopia. The identification of a novel and bidirectional ocular abnormality associated with *BMP4* truncations may provide new clues for elucidation of the developmental regulation of ocular size and shape.

## Materials and Methods

### Patient Recruitment and Data Collection

The probands with different eye disorders and their related family members were enrolled through the Pediatric and Genetic Clinic, Zhongshan Ophthalmic Center. Clinical data and peripheral blood samples were collected from these individuals. Prior to collection, all the participants or their guardians voluntarily signed informed content according to the tenets of the Declaration of Helsinki. This study was approved by the Institutional Review Board of Zhongshan Ophthalmic Center. Genomic DNA was extracted from the leukocytes within peripheral venous blood samples by following a previously reported method (Wang et al., 2010).

Each participating individual received a routine ophthalmologic examination. Additional specific ocular examinations were performed when required, including anterior segment photography, fundus photography, optic coherence tomography (OCT), electroretinogram (ERG), and scanning laser opthalmoscopy (SLO).

### Variant Detection and Evaluation

Whole-exome sequencing (Li et al., 2015) or target-exome sequencing (Wang et al., 2019) was performed on the genomic DNA from the 7,314 probands including 928 with early onset myopia, and 6,386 with other ocular conditions. After the detection of variants in *BMP4* from the exome sequencing data, the variants were filtered by multiple bioinformatic analytic steps. First, variants with low sequencing quality with a coverage of less than 5 were excluded. Second, synonymous and non-coding variants without effects on splicing site alterations, which were predicted by the Berkeley Drosophila Genome Project,^1^ were excluded. Third, through the evaluation of the minor allelic frequencies (MAFs) of variants based on the gnomAD database,^2^ variants with an MAF ≥ 0.01 were excluded. The remaining variants were evaluated by five *in silico* tools, including SIFT,^3^ Polyphen-2,^4^ PROVEAN,^5^ CADD,^6^ and REVEL.^7^ Finally, the variants were classified as potential pathogenic variants (PPVs) after comparison with the distribution of variants in our cohort and the gnomAD database.

The variants were further confirmed by Sanger sequencing. The online design program Primer3.0^8^ was used for primer design and the primer sequences are listed in Supplementary Table 1. Sanger sequencing validation including amplification, sequencing and target sequences analysis was performed following a previously described method (Chen et al., 2013). Then, the cosegregation analysis was conducted based on Sanger sequencing on genomic DNA from family relatives in these families.

### Statistical Analysis

IBM SPSS software version 26.0 (Amonk, NY: IBM Corp.) was applied to all statistical analyses in this study. The comparison of the frequency of truncation variants between in-house data related to early onset myopia and data in the gnomAD database was analyzed using the chi-square test or Fisher’s exact test. An in-house data comparison between the frequency of these truncation variants in patients with early onset myopia and the frequency of these truncation variants in patients with other eye conditions was also performed using the chi-square test or Fisher’s exact test. A *P*-value less than 0.05 was considered as statistically significant.

### Immunohistochemical Staining

To examine the BMP4 protein expression in the retinal tissue, immunohistochemical staining was performed on the human eyes of the donor who died of meningioma. The donor eyes were obtained from the Eye Bank of Guangdong Province. All the procedure was conducted following the Declaration of Helsinki and the written informed content was obtained from the donor before the study. The human eyes were fixed in the 4% paraformaldehyde and then embedded in paraffin. The paraffin-embedded eyes were cut into 4-μm-thick sections. Antigen retrieval was performed on the sections using high temperature (98°C) for 30 min and the sections were blocked with 5% normal goat serum. The primary antibodies, a mouse anti-BMP4 antibody (1:25; sc-12721; Santa Cruz) and a rabbit anti-PKC α antibody (1:500; sc-208; Santa Cruz), were used to incubate the sections. The secondary antibodies were Alexa Fluor 568-conjugated donkey anti-mouse IgG antibody (1:500; ab175472; Abcam) and Alexa Fluor 488-conjugated donkey anti-rabbit IgG antibodies (1:500; ab150073; Abcam) and DAPI (1:3000; 28718-90-3; Sigma-Aldrich) was used for the nuclear labeling. The images of stained sections were taken using the confocal microscope (*Zeiss LSM* 980, Carl Zeiss Microscopy GmbH, Jena, Germany).

## Results

### Mutation Analysis

**FIGURE 1:**
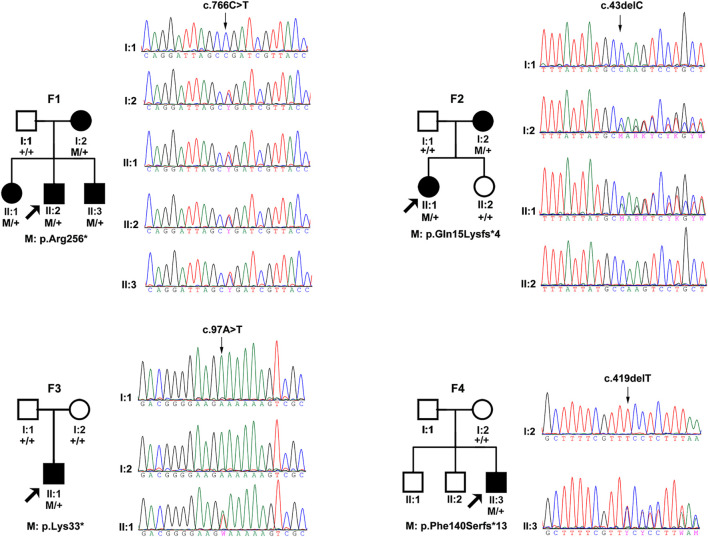
The pedigrees and sequencing chromatograms of the four families in this cohort with potential pathogenic truncations. The filled pattern represents affected individuals. The square pattern indicates males, while the circle pattern indicates female. M represents the mutated allele and + represents the normal allele. The family number is shown above the pedigree and the amino acid effect of the mutation is shown below the pedigree.

**FIGURE 2:**
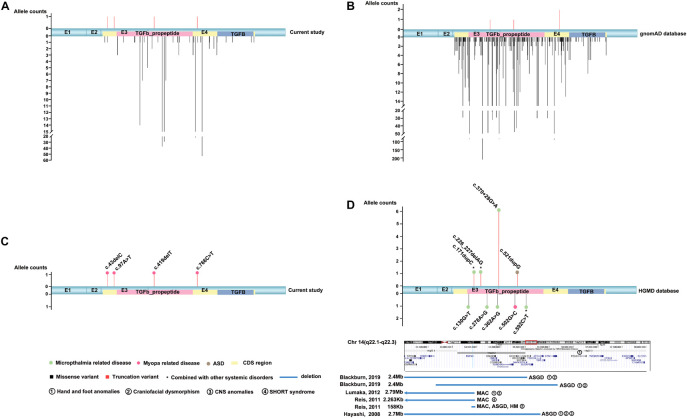
The distribution and frequency of *BMP4* variants in this cohort, the gnomAD database and the HGMD database. MAC, microphthalmia, anophthalmia, coloboma; ASGD, anterior segment dysplasia. **(A)** Diagram of the frequency and location of the detected *BMP4* variants in mRNA sequences from our cohort (accession number NM_001202.6). The truncation variants are shown above and the missense variants are shown below. Compared with allele counts of missense variants in this cohort, the truncation variants are extremely rare. **(B)** Schematic showing the distribution and frequency of *BMP4* variants in the gnomAD database. Truncation variants are rare in the general population (pLI = 0.96). **(C)** The distribution and frequency of potential pathogenic *BMP4* truncation variants in our in-house data. The filled square pattern represents the phenotype related to *BMP4* variants. The corresponding phenotype is shown above locations of the related variants. The potential pathogenic truncation variants in *BMP4* are enriched in patients with pathologic myopia. **(D)** The distribution and frequency of *BMP4* variants in previous studies. The truncation variants are shown above and the missense variants and large deletions are shown below. The blue rectangle patterns represent large deletions in *BMP4*. The position of references are indicated on the left of the pattern and the position of corresponding phenotypes is indicated on the right of the pattern. Based on the HGMD database, most reported disease-causing variants are associated with MAC and/or ASGD.

Totally, four novel truncation variants in *BMP4* were identified in four out of 7,314 unrelated probands with different eye conditions, including c.43delC:p.(Gln15Lysfs^∗^4), c.97A > T:p.(Lys33^∗^), c.419delT:p.(Phe140Serfs^∗^13), and c.766C > T:p.(Arg256^∗^) (Figure 1). These four truncations were novel and potential candidates of disease-causing variants since this type of variants were extremely rare and intolerant based on gnomAD database (pLI = 0.96) (Figure 2B). In the current study, interestingly, all the four probands with these *BMP4* truncations had early onset pathologic myopia, accounting for 4 of 928 probands with early onset high myopia, but in none of the 6,386 probands with other eye conditions (*P* = 2.58 × 10^–4^, Fisher’s exact test) (Figures 2A,C). Similarly, the frequency of *BMP4* truncations in probands with early onset high myopia is significantly higher compared with the frequency in gnomAD (*P* = 1.23 × 10^–7^, Fisher’s exact test) (Figures 2A–C). The four truncations in our cohort were confirmed by Sanger sequencing and completely cosegregated with pathologic myopia in all eight patients from the four families (Table 1 and Figure 1): the c.97A > T:p.(Lys33^∗^) was a *de novo* variant presented in Family F3; another nonsense variant [c.766C > T:p.(Arg256^∗^)] and two frameshift variants [c.43delC:p.(Gln15Lysfs^∗^4); c.419delT:p.(Phe140Serfs^∗^13)] were segregated with pathologic myopia in the remaining three families. Here, four novel BMP4 truncation variants exclusively segregated with pathologic myopia in eight patients from four unrelated families suggests a novel bidirectional role of BMP4 in the normal and abnormal development of the eye, since most *BMP4* variants were associated with microphthalmia-related disorders based on Human Gene Mutation Database (HGMD) database from previously studies (Bakrania et al., 2008; Hayashi et al., 2008; Reis et al., 2011; Lumaka et al., 2012; Huang X. et al., 2015; Blackburn et al., 2019; Nixon et al., 2019; Thanikachalam et al., 2020; Figure 2D).

**TABLE 1:** Clinical information of the patients with BMP4 truncation variants in this study.

Family	Nuleotide acid	Amino acid	Gender	Age (years)	Age (years)	BCVA		Axial length (mm)		Fundus	
ID	Change	Effect		Onset	At exam	OD	OS	OD	OS	OD	OS
F1-I:2	c.766C > T	p.Arg256*	F	EC	46	CF	CF	37.88	36.31	MA; PCA; TF	MA; PCA; TF
F1-II:1	c.766C > T	p.Arg256*	F	EC	23	0.40	0.40	29.38	31.44	PWSD; TF	DCA; PWSD; TF
F1-II:2	c.766C > T	p.Arg256*	M	EC	18	0.06	0.50	35.30	32.5	MA; PCA; PWSD; TF	DCA; PWSD; TF
F1-II:3	c.766C > T	p.Arg256*	M	EC	17	1.00	1.00	26.05	26.25	TF	TF
F2-I:2	c.43delC	p.Gln15Lysfs*4	F	NA	32	1.00	1.00	25.56	25.32	TF	TF
F2-II:1	c.43delC	p.Gln15Lysfs*4	F	3	7	0.80	0.80	29.30	27.72	DCA; PWSD; LD; TF	PWSD; LD; TF
F3-II:1	c.97A > T	p.Lys33*	M	EC	31	CF	HM	33.16	NA^$^	PS; DCA; TF	NA^$^
F4-II:3^#^	c.419delT	p.Phe140Serfs*13	M	3	6	0.20	0.40	32.08	31.19	PS; DCA; TF	PS; DCA; TF

*The variant nomenclature is based on the NCBI reference sequence for BMP4 transcript NM_001202.6. All variants are absent in HGMD database and gnomAD database. EC, early childhood; SE, Snellen equivalent; M, male; F, female; NA, not available; SA, affecting splicing acceptor; CF, counting fingers; HM, hand motion; TF, tessellated fundus; DCA, diffuse chorioretinal atrophy; MA, macula atrophy; PCA, patchy chorioretinal atrophy; PWSD, peripheral white spots degeneration; LD, lattice degeneration; BCVA, best corrected visual acuity.*

*^∗^Translation termination (stop) codon.*

*^$^The left eye of proband F3-II:1 has cornea opacity, cataract, and has been performed retinal photocoagulation due to retinal detachment. The fundus photo and axial length of the left eye from this patient is unavailable.*

*^#^Extra-ocular features were only observed in one of the eight patients (F4-II:3), who had tooth malformation, broad nasal bridge, and hyperextensible joints.*

### Clinical Characteristics

**FIGURE 3:**
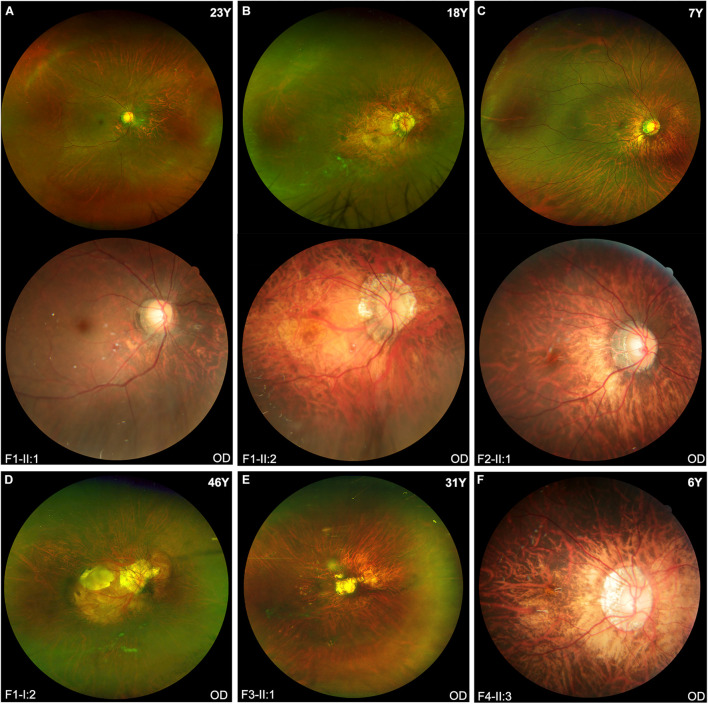
The fundus images of the probands in four families. **(A,B,D)** Typical pathologic myopia fundus changes in the right eyes of three patients from Family F1. All patients carried the same heterozygous truncation variant c.766C > T (p. Arg256*). Fundus photography of Patients F1-II:1 and F1-II:2 shows the ultra-widefield fundus imaging above and traditional fundus imaging of the posterior pole below. **(C)** The fundus images from the right eye of Patient F2-II:1 show diffuse chorioretinal atrophy and white spots in the peripheral retina. **(E)** Scanning laser ophthalmoscopy imaging of Patient F3-II:1 appears diffuse chorioretinal atrophy surrounding the optic disc region, patchy atrophy and posterior staphyloma. **(F)** Posterior staphyloma and diffuse chorioretinal atrophy around the optic disk are observed in the fundus photo of Patient F4-II:3.

**FIGURE 4:**
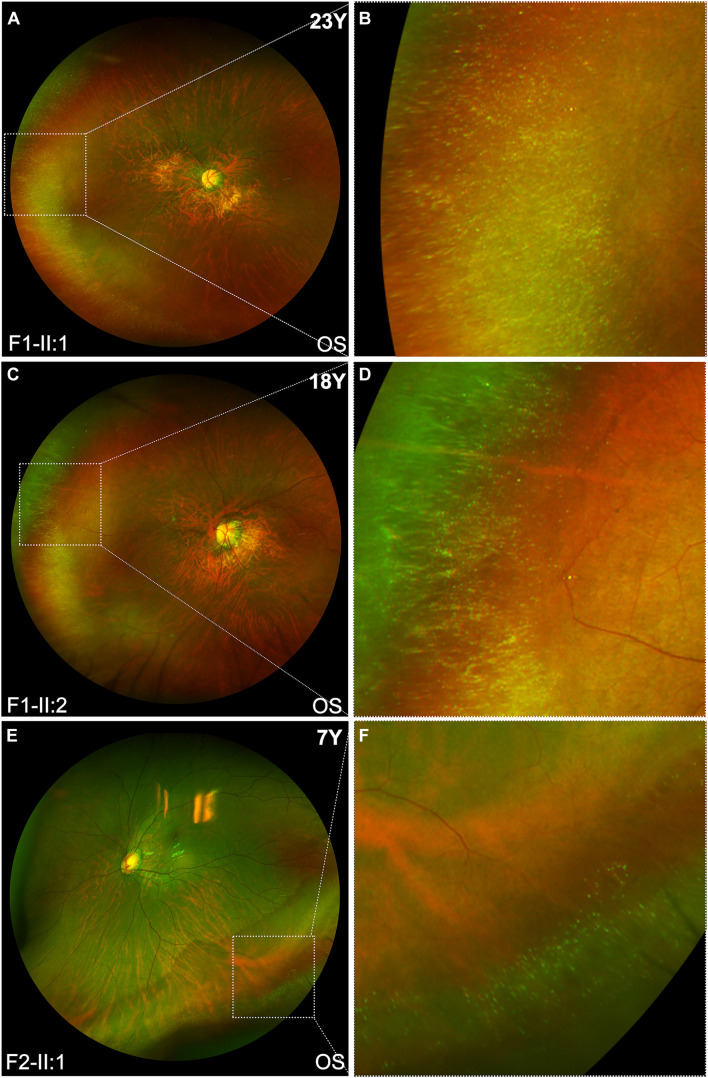
The characteristic snow-grain like retinopathy in peripheral fundus of patients with *BMP4* truncation variants. **(A,C,E)** Representative ultra-widefield fundus imaging of the left eyes from three patients: F1-II:1, F1-II:2, and F2-II:1. **(B,D,F)** The corresponding magnified image demonstrates that white dots in peripheral retina of the three patients closely resemble snow grains.

The clinical features of eight patients with novel *BMP4* truncation variants were summarized in Table 1. Extra-ocular features were only observed in one of the eight patients, i.e., proband F4-II:3, who had tooth malformation, broad nasal bridge, and hyperextensible joints. Of the eight patients, the four probands were identified to have high myopia due to poor vision before school age. Subsequent systemic ocular examination on the probands and family members revealed pathologic myopia in eight patients from the four families. The best-corrected visual acuity (BCVA) among eight patients ranged from counting fingers (CF) to 1.00 (Snellen equivalent) and the mean BCVA was 0.45. The average axial length of eight patients was 30.62 mm (range, 25.32–37.88 mm). Additionally, the axial length of all patients but one family member (F2-I:2) was more than 26.00 mm. The axial length of F2-I:2 was 25.56 mm in the right eye and 25.32 mm in the left eye, her fundus changes showed a trend toward pathologic myopia. The fundus images of these patients demonstrated typical fundus changes for pathologic myopia, including tessellated fundus, posterior staphyloma, macula atrophy, patchy or diffuse chorioretinal atrophy, and peripheral chorioretinal degeneration (Figure 3). Interestingly, unique fundus changes were clearly observed in peripheral retinas of three patients from two families (F1-II;1, F1-II:2, and F2-II:1), i.e., numerous small white spots in the peripheral retina displayed a “snow grain” appearance (Figure 4). This characteristic phenotype has not been described previously in pathologic myopia so that it may be considered as a unique sign of pathologic myopia related to *BMP4* truncation variants. The fundus autofluorescence and ERG test result were available from both eyes from the proband F1-II:2, demonstrating moderate reduction of rod and cone responses on ERG examination and relatively preserved autofluorescence with non-specific minor changes in the posterior and mid-peripheral retina (Supplementary Figure 1). Additionally, difference in severity of pathologic myopia between the two eyes was observed in all patients (Figures 3, 4), but such difference was relatively mild as compared with pathologic myopia due to mutations in genes responsible for Stickler syndrome or FEVR (unpublished data). The OCT scans of the four probands illustrated optic nerve fiber layer thinning and choroid atrophy (Supplementary Figures 2A–D). The white vitreous strands resembling gossamer anomalies were observed in anterior vitreous cavity in patients from two families (F1, F2) (Supplementary Figure 2E).

## Discussion

Previously, mutations in *BMP4* were known to cause microphthalmia-related disorders (Bakrania et al., 2008; Reis et al., 2011). On the contrary, in the current study, four novel truncation variants in *BMP4* were identified as PPVs in eight patients with pathologic myopia from four families. Our novel findings were supported by the following lines of evidences: (1) As described above, the truncation variants in *BMP4* were extremely rare and intolerant in general population (gnomAD database, pLI = 0.96). Although the few *BMP4* truncation variants have been reported are related to ocular phenotypes based on HGMD database, the loss of function variant has been identified as a disease-causative mutation related to ocular or systemic disorders. (2) As previously mentioned, the four *BMP4* truncation variants considered PPVs, were highly enriched in 928 patients with early onset high myopia in this cohort. In contrast, none of potential pathogenic *BMP4* truncation variants were identified in 6,386 patients with other eye conditions. Essentially, the clinical evidence supports that these four truncation variants contribute to phenotype—pathologic myopia. This point reflects that truncation variants in *BMP4* are highly related to pathologic myopia. (3) None of these four *BMP4* truncation variants were present in databases. These variants segregated with pathologic myopia in all families in this cohort. (4) In the same pedigree, all individuals with same *BMP4* truncation variant exhibited a similar ocular phenotype. For example, in the family F1, the fundus images of mother and three children with same variant showed typical pathologic myopia fundus changes based on the Meta-Analysis for Pathologic Myopia (META-PM) classification (Ohno-Matsui et al., 2015). The same snow-grain degeneration in the peripheral retina was observed in the proband’s sister (F1-II:1) and the proband (F1-II:2). (5) In our study, the BMP4 protein expression mainly located in the inner nuclear layer and inner plexiform layer of adult human retina (Supplementary Figure 3), indicating that BMP4 might play pivotal role in visual and retinal development. (6) Previous genome-wide association studies on myopia identified that one of new genetic associations in European population was near the location of *BMP4* (Kiefer et al., 2013). Based on the above evidences, the *BMP4* truncation variants are suggested to cause pathologic myopia.

Interestingly, the novel phenotype related to *BMP4* truncations observed in our cohort is opposite to previously reported phenotypes. In previous studies, variants in *BMP4* have been mainly reported to be associated with microphthalmia, anophthalmia, coloboma (MAC) (Bakrania et al., 2008; Reis et al., 2011) and anterior segment dysgenesis (ASGD) (Takenouchi et al., 2013) in human. As a member of the BMP family and transforming growth factor-β (TGF-β) superfamily, *BMP4* is known to play the critical role in the embryonic development (Hogan, 1996). In the eye, the *BMP4* gene has been reported to be engaged in normal ocular morphogenesis involving lens induction (Huang J. et al., 2015), ciliary body formation (Rausch et al., 2018), and retinal development (Murali et al., 2005; Maruyama et al., 2006; Thompson et al., 2019). Previous studies and our current data indicate that *BMP4* may play a bidirectional role in developmental regulation of ocular shape and size. Recently, genome-wide association studies based on a large population of individuals of European and Asian ancestry showed that the same set of variants shared contributions to the genetic risk of high myopia, low myopia and hyperopia (Tideman et al., 2021) in multifactorial manner. In fact, contrary phenotype due to a *BMP4* truncation mutation was reported in one family, where the proband had unilateral anophthalmia, small cornea, and iris and chorioretinal coloboma, while his three family members with the same mutation had high myopia (Bakrania et al., 2008). Except for *BMP4*, opposite ocular phenotypes have been associated with mutations in other genes, such as *FZD5*, in which individuals with the same truncation variant or different eyes of the same individual exhibited either microphthalmia/uveal coloboma or high myopia (Jiang et al., 2021). Similar situation has been observed for *PAX6*, a gene known to cause aniridia when mutated, in which several single nucleotide polymorphisms are significantly associated with myopia (Hammond et al., 2004). Besides, similar situation also occurs in extraocular system, for example, variants in *M4CR* related to a gain of function tended to result in a lower risk of obesity while *M4CR* variants related to a loss of function contributed to a higher risk of obesity (Lotta et al., 2019). These evidences raise the hypothesis that some genes, such as *BMP4*, may be involved in bidirectional rather than unidirectional control of early ocular development. The mechanism of bidirectional regulation remains unknown and requires further studies.

In our current study, comparative exome sequencing, mutation-specific phenotypic clustering, cosegregation in multiple families, rarity and intolerant of truncations in general population, all provides strong evidence to support that truncation variants in *BMP4* contribute to a novel phenotype of pathologic myopia. The snow-grain degeneration in the peripheral retina may be a characteristic sign specific for *BMP4*-related pathologic myopia. Further studies are expected to confirm our findings and to elucidate the underlying molecular mechanism of bidirectional regulation of eye development.

## Data Availability Statement

The datasets presented in this study can be found from the following link: https://bigd.big.ac.cn/gsa-human/browse/HRA001597. The accession number is HRA001597.

## Ethics Statement

The studies involving human participants were reviewed and approved by Institute Review Board of the Zhongshan Ophthalmic Center. Written informed consent to participate in this study was provided by the participants’ legal guardian/next of kin.

## Author Contributions

XX, SL, QZ, XJ, and LZ contributed to the patient recruitment and diagnosis. XX, QZ, XJ, YJ, JO, XL, YW, and LZ collected the clinical records. XX, SL, PW, and QZ performed the whole-exome analysis and targeted-exome sequencing. QZ contributed to the conception and design of this study and revised thoroughly the manuscript. WS, XX, PW, SL, QZ, and YJ performed the statistical analysis. YJ confirmed the variants by Sanger sequencing and family segregation analysis and wrote the first draft of the manuscript. All authors reviewed the manuscript and approved for submission.

## Conflict of Interest

The authors declare that the research was conducted in the absence of any commercial or financial relationships that could be construed as a potential conflict of interest.

## Publisher’s Note

All claims expressed in this article are solely those of the authors and do not necessarily represent those of their affiliated organizations, or those of the publisher, the editors and the reviewers. Any product that may be evaluated in this article, or claim that may be made by its manufacturer, is not guaranteed or endorsed by the publisher.

## Footnotes

1
https://www.fruitfly.org/

2
https://gnomad.broadinstitute.org/

3
http://sift.jcvi.org/

4
http://genetics.bwh.harvard.edu/pph2/

5
http://provean.jcvi.org/seq_submit.php

6
http://cadd.gs.washington.edu

7
https://sites.google.com/site/revelgenomics/

8
http://primer3.ut.ee/
