# Supplementary material for: Novel BMP4 Truncations Resulted in Opposite Ocular Anomalies: Pathologic Myopia Rather Than Microphthalmia
Source: Front Cell Dev Biol. 2021 Dec 1;9:769636. doi: 10.3389/fcell.2021.769636 (PMC8672680; doi:10.3389/fcell.2021.769636)
Supplement: Supplementary file 1 [file Data_Sheet_1.PDF]

Supplementary Figure S1 | The optical coherence tomography (OCT) scans and ocular anterior segment photographs of patients in this cohort with *BMP4* truncation variants. (A-D) The OCT scans of four patients (F1-I:2, F1-II:2, F2-II:1, F4-II:3) demonstrated optic nerve fiber layer thinning and choroid atrophy. **(E, F)** The anterior segment photos of left eyes from two patients in Family F1 strands floating in the vitreous cavity.

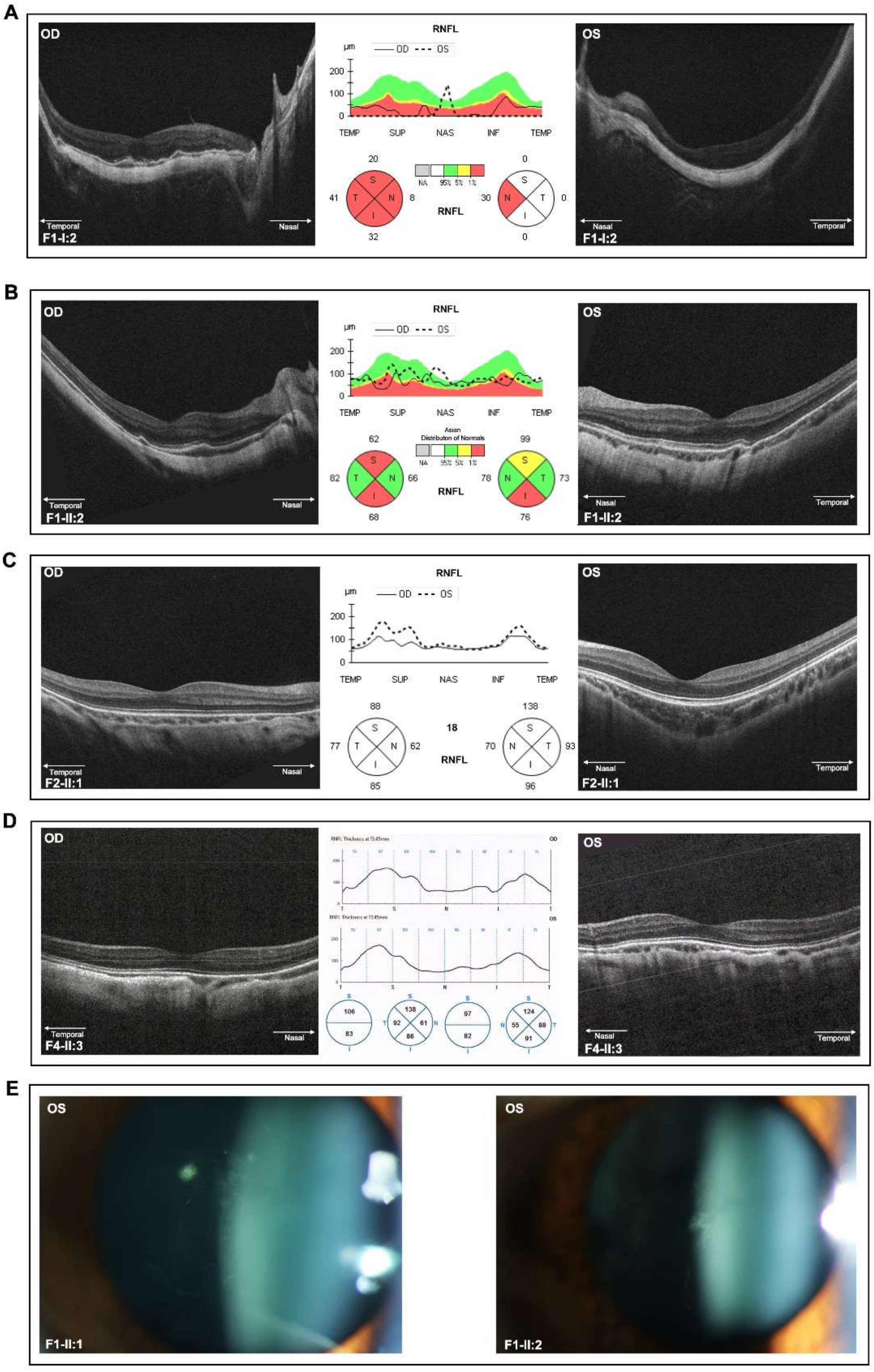

Supplementary Figure S2 | The immunostaining for BMP4 protein expression in human retinal. (A) The representative images of human retina showed that the staining for BMP4 mainly located in the inner nuclear layer and inner plexiform layer, **(B)** whereas no positive signal could be observed in the negative controls. The staining for α-PKC indicated the location of rod bipolar cells. NC, negative controls; GCL, ganglion cell layer; IPL, inner plexiform layer; INL, inner nuclear layer; OPL, outer plexiform layer; ONL, outer nuclear layer; All scale bars represent 10 μm.

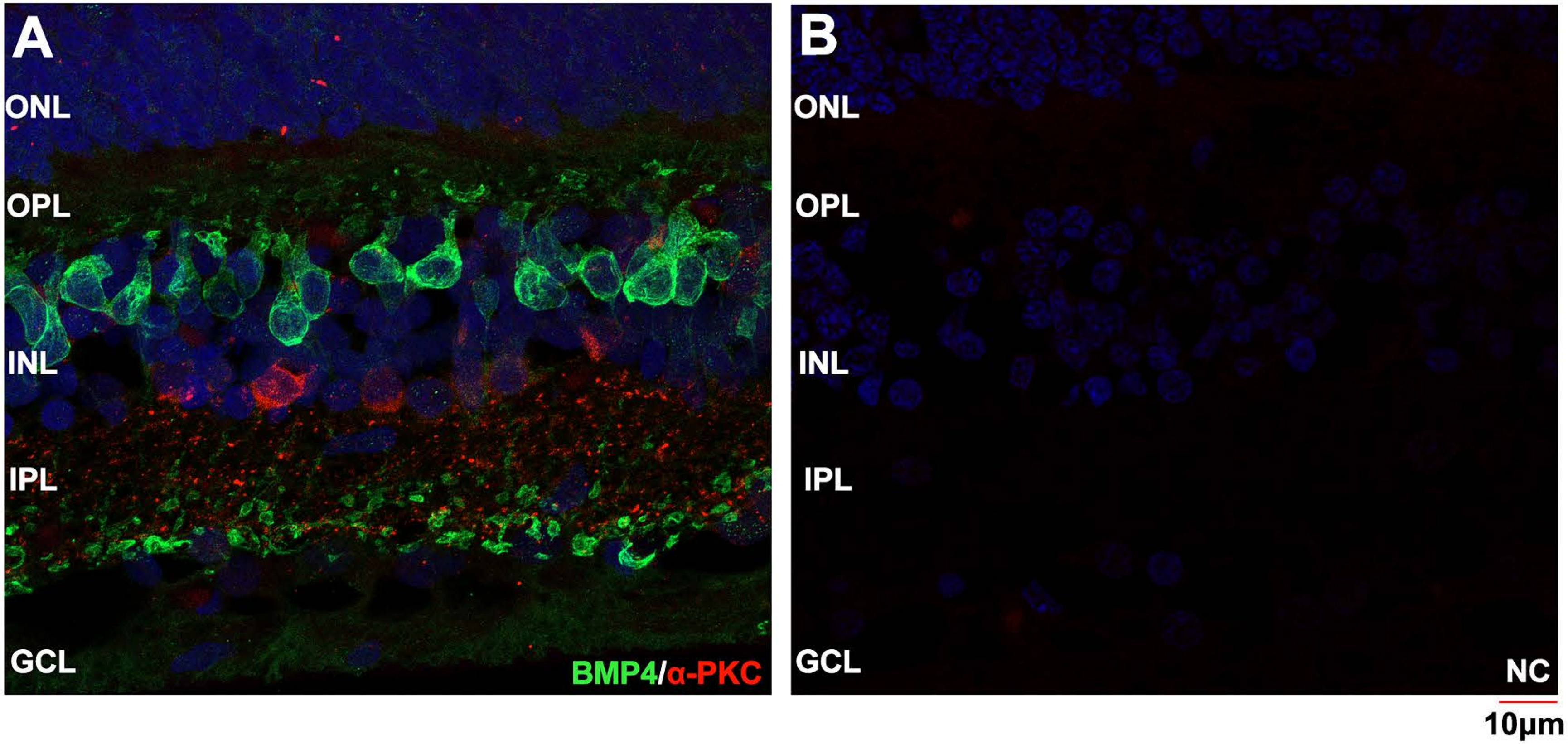

Supplementary Figure S3 | The visual field examination result and fundus autofluorescence image of the proband (F1-II:2) **(A)** The 24-2 visual field examination result showed the slightly enlargement of blind spot in the left eye. **(B)** The fundus autofluorescence image showed nonspecific minor changes in the posterior and mid-peripheral retina of the left eye.

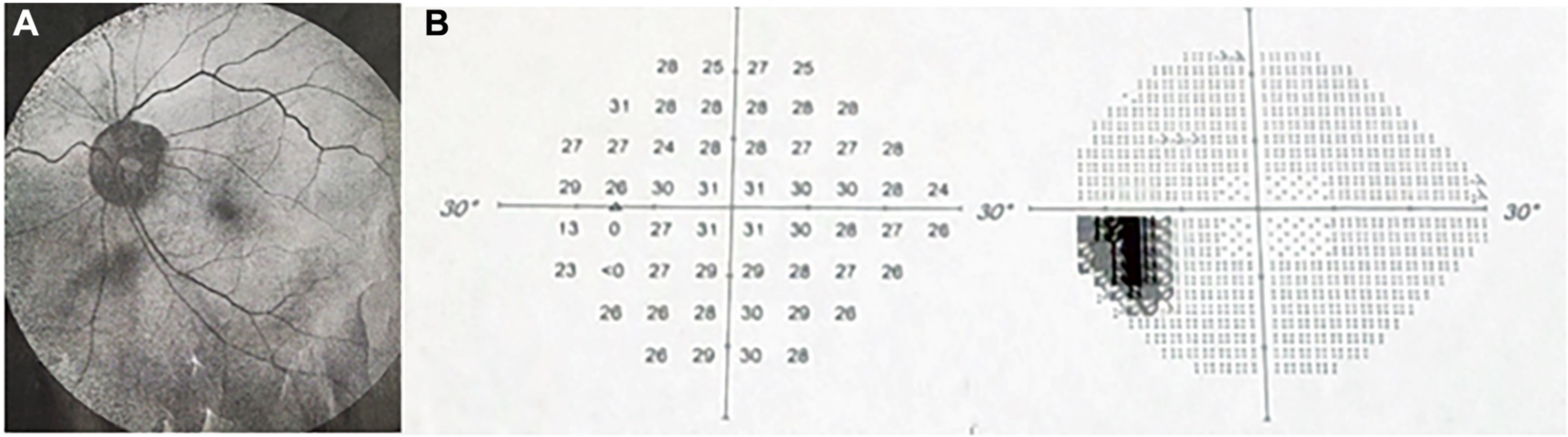

Supplementary Table S1: PCR and sequencing primers for BMP4 (accession number NM_001202.6)

Exon	Forward primer (5'-3') Reverse primer (5'-3') (amplication length [bp])	Amplication length [bp]	PCR cycling conditions					
			Pre-denaturation	Denaturation	Annealing	Extension	Cycles	Final extension
**3**	CCTGGTAACCGAATGCTGAT<br>GGACTGGGGCTTTGATGTAA	472	5min at 95°	30s, 95°	30s, 60°	40s, 72°	35x	10min, 72°
**3**	GGTGGTGTGAGGGAGAAGAC<br>ACTGGGGGAAGAGACTGACC	556	5min at 95°	30s, 95°	40s, 60°	40s, 72°	35x	10min, 72°
**4**	CTGGTCCACCACAATGTGAC<br>GGCTTTGGGGATACTGGAAT	506	5min at 95°	30s, 95°	40s, 60°	40s, 72°	35x	10min, 72°
**4**	TTTTCCCCCAGTAGGTTTCC<br>CGATCGGCTAATCCTGACAT	538	5min at 95°	30s, 95°	40s, 60°	40s, 72°	35x	10min, 72°
